# Mortality burden of malignant neoplasms of the corpus uteri in Mexico: an analysis of trends from 2000 to 2022

**DOI:** 10.1590/1980-549720260001

**Published:** 2026-01-30

**Authors:** Eugenia Flores-Alfaro, Felipe Adrián Encarnación-López, Gabriela Elizabeth Campos-Viguri, Maria Elena Moreno-Godínez, Ana Karen Herrera-Vargas, Marco Antonio Ramírez-Vargas

**Affiliations:** I Universidad Autónoma de Guerrero, Facultad de Ciencias Químico-Biológicas, Laboratorio de Investigación en Epidemiología Clínica y Molecular – Chilpancingo (GRO), México.; II Universidad Autónoma de Guerrero, Facultad de Ciencias Químico-Biológicas, Laboratorio de Toxicología y Salud Ambiental – Chilpancingo (GRO), México.; III Unidad Morelos-Instituto Politécnico Nacional, Centro de Investigación en Ciencia Aplicada y Tecnología Avanzada – Cuernavaca, Morelos, México.

**Keywords:** Endometrial neoplasms, Mortality, Mexico, Uterine neoplasms, Neoplasias do endométrio, Mortalidade, México, Neoplasias uterinas

## Abstract

**Objective:**

The aim of this study was to analyze temporal trends in malignant neoplasms of corpus uteri mortality in México from 2000 through 2022.

**Methods:**

Certificates of death were analyzed. The age-standardized mortality rate (ASMR), the number of years of life lost, according to the federal state, was estimated. The changes in temporal trends were analyzed using joint point regression.

**Results:**

The ASMR from malignant neoplasms of the corpus uteri was estimated at 2.5 (95% confidence interval [CI] 2.2–2.8), and the annual percentage change from 2000 to 2018 was 4.32. The age group of 60–65 years was most affected.

**Conclusion:**

This report indicates an increase in the ASMR from malignant neoplasms of the corpus uteri. It also suggests the need for the development of public health laws focusing on the early diagnosis and prevention of uterine cancer.

## RESUMO

**Objetivo:**

Analisar as tendências temporais da mortalidade por neoplasia maligna do corpo do útero no México entre 2000 e 2022.

**Métodos:**

Foram analisados certificados de óbito. Estimaram-se a taxa de mortalidade padronizada por idade e o número de anos de vida perdidos de acordo com o estado federativo. As mudanças nas tendências temporais foram avaliadas por meio de *joinpoint regression*.

**Resultados:**

A taxa de mortalidade padronizada por idade para neoplasia maligna do corpo do útero foi estimada em 2,5 (intervalo de confiança de 95% 2,2–2,8), com variação percentual anual de 4,32 entre 2000 e 2018. O grupo etário de 60 a 65 anos foi o mais afetado.

**Conclusão:**

Este estudo indicou aumento na taxa de mortalidade padronizada por idade para neoplasia maligna do corpo do útero. Os achados sugerem a necessidade do desenvolvimento de políticas públicas voltadas ao diagnóstico precoce e à prevenção do câncer uterino.

## INTRODUCTION

Gynecological cancers are malignant tumors affecting the female reproductive system. These cancers include vaginal, vulvar, cervical, uterine, and ovarian cancer. Gynecological cancer distribution varies according to country, age group, and ethnicity. A global analysis of the annual percentage change (APC) (1990–2021) in gynecological cancers showed that malignant neoplasms of the corpus uteri exhibit an increase (0.45), followed by ovarian cancer (0.06), and cervical cancer demonstrate a reduction (-0.39). However, the APC and incidence associated with gynecological cancers vary between countries; for example, high-income countries show a constant increase in the incidence of malignant neoplasms of the corpus uteri with a reduction in cervical cancer, while in low-income countries, the incidence of cervical cancer is high and constant, with a non-significant increase in malignant neoplasms of the corpus uteri^1^.

In 2019, it was estimated that malignant neoplasms of the corpus uteri were the second leading cause of death related to gynecological cancers in the world^2^. The risk factors related to gynecological cancer include a complex interaction between biological factors (such as obesity, age of menarche and menopause, parity, and genetic background), environmental factors (such as the use of hormonal replacement therapy, the use of oral contraception therapy, and physical activity), sociocultural factors (such as diet and job), and healthcare accessibility factors^3^ that could result in differential incidence and mortality rates.

In México, malignant neoplasms are the leading cause of registered death in women^4^. Malignant neoplasms of the corpus uteri are the second most frequent gynecological cancer in México. In 2020, the age-standardized mortality rate (ASMR) was estimated at 7.6 per 100,000 inhabitants; endometrial cancer is the most frequent subtype of malignant neoplasms of the corpus uteri in México^5^. Moreover, risk factors for multiple malignant neoplasms of the corpus uteri converge in México, including an increase in life expectancy and an increase in the incidence/prevalence of obesity^6^. However, the information about the distribution of mortality related to malignant neoplasms of the corpus uteri in México is not available. This work aimed to analyze mortality trends in malignant neoplasms of the corpus uteri in México from 2000 through 2022.

## METHODS

We performed a time series ecological study employing an epidemiologic dataset from "Secretaría de Salud" (México's Health Secretary) using the "Sistema estadístico epidemiológico de las defunciones" (Epidemiological Statistical System of Deaths)^7^. We used data of registered deaths attributed to malignant neoplasms of the corpus uteri from 2000 through 2022 in México. The cases were identified employing the International Classification of Diseases and Related Health Problems, 10th Revision (ICD-10)^8^. The number of deaths related to malignant neoplasms of the corpus uteri was identified for the C54 code. Moreover, the anatomical parts of cancer localization that included isthmus uteri (C54.0), endometrium (C54.1), myometrium (C54.2), fundus uteri (C54.3), corpus uteri, and unspecified (C54.9) were considered. Furthermore, the registered death age (quinquennial age groups), the state where the patient lived, and the year of death registration were considered. The population estimate was obtained from the National Institute of Geography and Statistics (INEGI)^9^ and National Population Council (CONAPO)^10^.

### Statistical analysis

The annual mortality rate per 100,000 inhabitants^11^, and the ASMR per 100,000 inhabitants^12^ were calculated by Equations 1, 2 and 3.


(1) $$Annual\;mortality\;rate\text{:}\frac{No.of\;deaths\;due\;to\;malignant\;neoplasms\;of\;the\;corpus\;uteri}{No\text{.}of\;persons\;in\;the\;population\;at\;midyear}X100,000)$$

(2) $$(\sum_{i}\frac{d_{i}w_{i}}{y_{i}};d_{i}:\;No\text{.}of\;deaths\;;w_{i}:Standard\;world\;population\;according\;to\;the\;WHO\text{;}$$

(3) $$y_{i}:person-years\;at\;risk)$$

In addition, the age-standardized years of life lost rate (ASYR) per 100,000 inhabitants were estimated by Equation 4.


(4) $$(ASRY_{\left(c,s,t\right)}=\sum_{a}\begin{array}{c} YLL_{rate\;(c,a,t)}\times W_{\left(a\right)};YLL_{rate}:\text{years of life lost rate due to malignant neoplasms of the corpus uteri; c: malignant neoplasms of the corpus uteri; a: age; t: period}) \end{array}$$

The temporal trends in mortality were analyzed through joint point regression. This regression considers a Poisson distribution log(*y*_i_) = ε[*y*_i_|*x*_i_] + ε_i_. The period from 2000 to 2022 is reflected as *x*_i_; the *y*_i_ corresponds to annual rates. The ε_i_ symbolizes residuals for the *i*-th period, and the ε[*y*_i_|*x*_i_] reveals the mean from regression. The mean is calculated following a consecutive linear segment (n+1) over the period (from 2000 "a" to 2022 "b"): [*a*,*b*]: ε[*y*_i_|*x*_i_] = β_0_ + β_1_ *x*_i_ + δ_1_ (*x*_i_ – τ_1_)+ + … + δ_n_ (*x*_i_ – τ_n_)^+^. The result from the joint point regression shows that the trend line slope change could be considered statistically significantly distinct from zero^13^. The interpretation of changes in temporal trends is closely tied to the values of the slope and its p-value. A value of slope>0 with a p<0.05 indicates an ascending trend. Conversely, the value of slope<0 with a p<0.05 indicates a descending trend. Moreover, a slope with a p>0.05 indicates a stable trend^14^. Finally, we reported the results considering the best model that included a temporal stratification that decreases the error from the joint point regression; the best model was selected considering the weighted Bayesian information criterion^15^. The joinpoint analysis was performed using the "Joinpoint Regression Program version 4.9.1.0"^16^.

Furthermore, we grouped the federal states according to mesoregions; the mesoregions are composed of states with a similar distribution of economic indicators. The mesoregions were constituted according to reports for the Federal Expenditure Budget. Five mesoregions were confirmed: South-Southeast (Campeche, Chiapas, Guerrero, Oaxaca, Puebla, Quintana Roo, Tabasco, Veracruz, and Yucatán); Central-West (Aguascalientes, Colima, Guanajuato, Jalisco, Michoacán, Nayarit, San Luís Potosí, and Zacatecas); Central (Ciudad de México, Estado de México, Hidalgo, Morelos, Querétaro, and Tlaxcala); Northeast (Chihuahua, Coahuila, Durango, Nuevo León, and Tamaulipas), and Northwest (Baja California, Baja California Sur, Sinaloa, and Sonora)^17^. We considered a p<0.05 as statistically significant.

#### Data availability statement:

The data that support the findings of this study are available from the corresponding author [MARV] upon reasonable request.

## RESULTS

**Figure 1:**
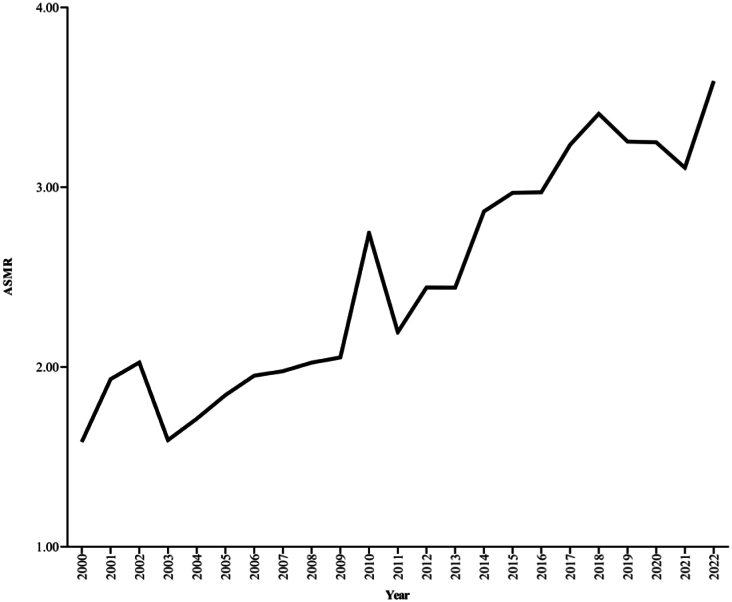
Age-standardized mortality rate per 100,000 inhabitants from malignant neoplasms of the corpus uteri.

A total of 12,079 deaths were attributed to malignant neoplasms of the corpus uteri in México from 2000 through 2022. The highest peak of ASMR per 100,000 inhabitants was registered in 2022 (Figure 1). The average of ASMR from 2000 to 2022 was estimated to be 2.5 (95% confidence interval [CI] 2.2–2.8). Non-significant differences were found between each mesoregion's national and average mortality rates (p>0.05). The distribution of ASMR per 100,000 inhabitants according to early or late occurrences of corpus malignant neoplasms of the corpus uteri is shown in Figure 1.

**Figure 2:**
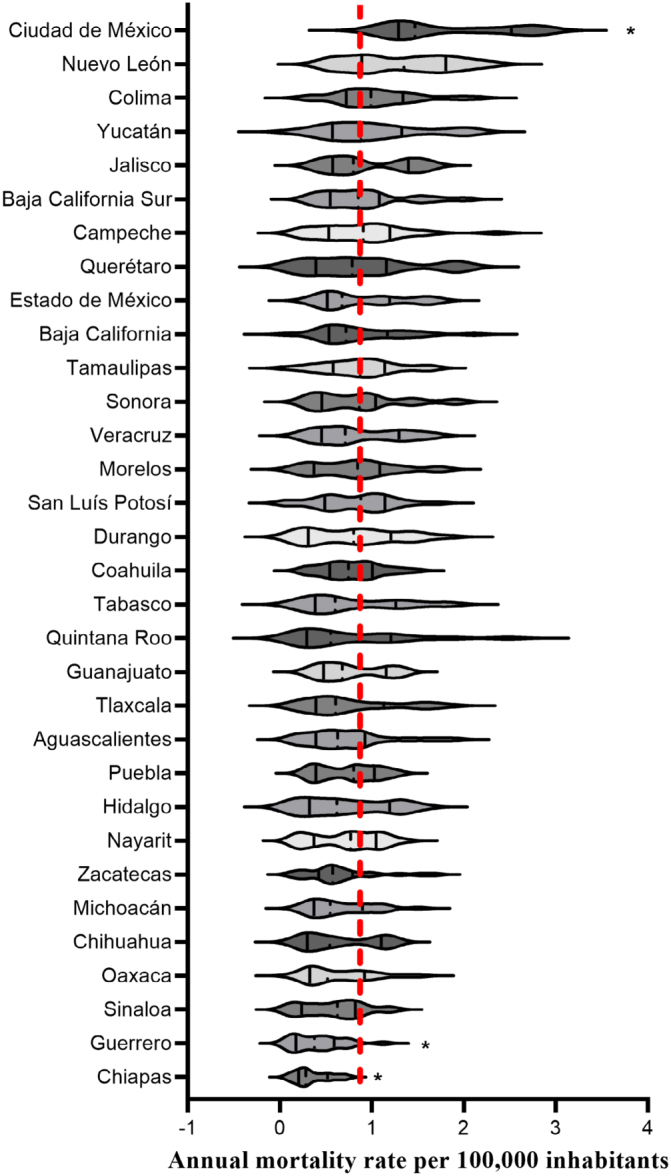
Comparison between ASMR for the federal state. Violin plots represent the distribution of ASMR per 100,000 inhabitants in the federal state. The national average is represented in the dotted red line.

Figure 2 shows each state's mortality rate per 100,000 inhabitants (from 2000 to 2022). Compared to the national average, Chiapas and Guerrero states showed the lowest mortality rate per 100,000 inhabitants, and Ciudad de México showed the highest.

*Statistically significant difference compared with the national average (p<0.05).

The frequency of malignant neoplasms of the corpus uteri, according to the uterine anatomic site, was as follows: endometrium (97.13%), unspecified corpus uteri (2.24%), isthmus uteri (0.32%), myometrium (0.22%), and fundus uteri (0.09%). The same distribution was identified in all mesoregions.

**Figure 3:**
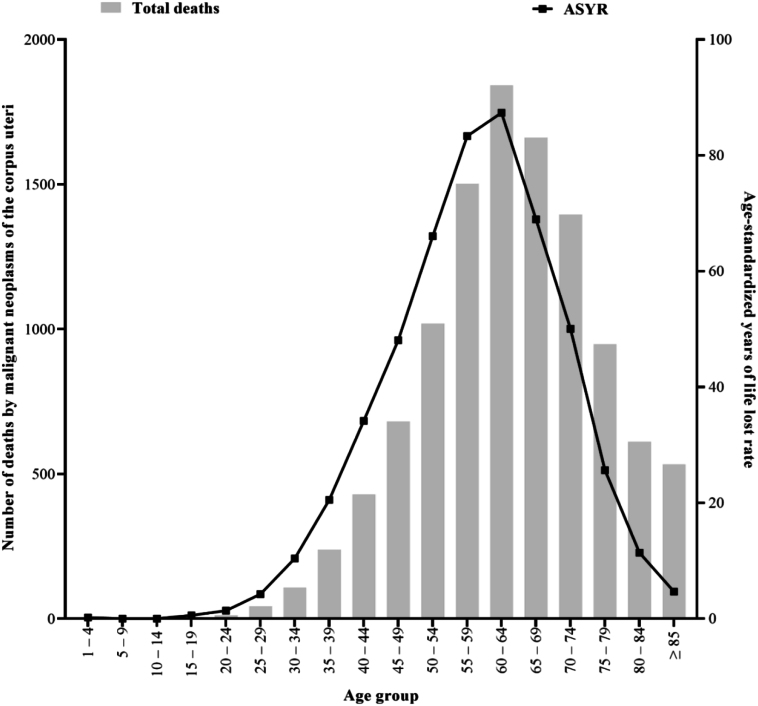
Age-standardized years of life lost rate per 100,000 inhabitants.

A total of 20,469 years of life lost rate per 100,000 inhabitants was attributed to malignant neoplasms of the corpus uteri in México from 2000 through 2022. Figure 3 represents the distribution of ASYR according to the quinquennial age group. The ASYR follows a unimodal distribution (negatively skewed curve shape) with a peak at the 60–64 group. The distribution of the number of deaths according to age group represents a gradual increase in 35–39 years (middle-aged adults), with a peak at 60–64 years (older-age adults) and a decrease concerning the increase in age (≥65 years). This distribution suggests that malignant corpus uteri neoplasms mainly affect older age groups in México (Figure 3). Similar distributions were observed in all mesoregions.

The temporal trend analysis of the ASMR of 100,000 inhabitants from malignant neoplasms of the corpus uteri in México is presented in Tables 1 and 2. Table 1 shows an increase in APC (4.32) from 2000 to 2018 and a non-significant change in APC from 2018 to 2022. The South-Southeast region showed the highest APC from 2007 to 2022; the increase was significant (APC=9.88, p<0.05). The central-west, Centro, and northwest regions showed a similar APC (6.07, 6.34, and 6.7, respectively) from 2000 to 2022; in all cases, the increase was significant (p<0.05). The northeast region showed an APC of 7.56 (p<0.05). The APC in the analysis of ASMR for 100,000 inhabitants is presented in Table 2. The national average annual percentage change (AAPC) was estimated at 3.14, and the mesoregions showed a similar AAPC (p<0.05).

**Table 1:** Annual percent change of age-standardized mortality rate from malignant neoplasms of the corpus uteri in Mexico (from 2000 through 2022).

Region	Segment	Period	APC	95%CI	p-value
National	1	2000–2018	4.32	3.74–5.79	<0.0001
	2	2018–2022	-2	-7.37–0.99	0.18
South-Southeast	1	2000–2007	-0.68	-26.55–7.57	0.76
	2	2007–2022	9.88	7.37–21.65	0.03
Central-West	1	2000–2022	6.07	5.41–6.84	<0.0001
Centro	1	2000–2022	6.34	5.23–7.63	<0.0001
Northeast	1	2000–2017	7.56	6.76–11.59	0.003
	2	2017–2022	2.56	-7.28–5.88	0.42
Northwest	1	2000–2022	6.7	5.85–8.1	<0.0001

APC: annual percentage change; 95%CI: 95% confidence interval.

**Table 2:** Average annual percentage change of age-standardized mortality rate from malignant neoplasms of the corpus uteri in Mexico (from 2000 through 2022).

Region	AAPC	95%CI	p-value
National	3.14	2.63–4.07	<0.0001
South-Southeast	6.4	4.47–8.99	<0.0001
Central-West	6.07	5.41–6.84	<0.0001
Centro	6.34	5.23–7.63	<0.0001
Northeast	6.4	5.56–7.54	<0.0001
Northwest	6.7	5.85–8.1	<0.0001

APC: annual percentage change; 95%CI: 95% confidence interval.

The APC or AAPC from the years of life lost (YLL) or age-standardized YLL rate (ASYLR) is presented in Table 3. The national AAPC from YLL and ASYLR were calculated as 4.15 and 5, respectively; the changes in AAPC were significant. Moreover, the changes in APC from YLL showed an increase of 4.86 (from 2000 to 2019; p<0.05) and an increase of 5.89 (from 2000 to 2017; p<0.05).

**Table 3:** Annual percentage change or average annual percentage change of YLL and ASYLR in Mexico (from 2000 through 2022).

Parameter	Segment	Period	APC	95%CI	p-value
YLL	1	2000–2019	4.86	4.43–9.62	<0.001
	2	2019–2022	-0.23	-5.45–3.71	0.98
ASYLR	1	2000–2017	5.89	5.89–5.25	<0.0001
	2	2017–2022	2.03	-2.25–4.4	0.18
**Parameter**	**Full range**		**AAPC**	**95%CI**	**p-value**
YLL	2000–2022		4.15	3.73–5.74	<0.0001
ASYLR	2000–2022		5	4.48–7.38	<0.0001

YLL: Years of life lost rate; ASYLR: Age-standardized years of life lost rate per 100,000 inhabitants

## DISCUSSION

Gynecological cancers are considered the leading cause of mortality in the world. Risk factors for developing gynecological cancers include complex interactions between several factors that include biological (such as genetic background, age, and comorbidities that include viral infections, obesity, and others), sociocultural, and health care accessibility that result in differential mortality for each country. Under this context, we performed an epidemiological description of the mortality trends related to malignant neoplasms of the corpus uteri in México from 2000 through 2022. The results showed that Mexican women aged≥65 years old are most affected. No differences were observed in the ASMR between mesoregions. Furthermore, an increase in the AAPC in ASMR was observed in México.

Malignant tumors are a key cause of death in the world, and cancer in the uterine corpus was the seventh most prevalent cancer that affected females in 2022. A total of 183,093 deaths associated with cancer in the uterine corpus are projected in 2050^18^. In this context, malignant tumors are considered a key cause of mortality in México. In the preliminary report of national statistics of death for 2024, cancer was identified as the third cause of death for both sexes^4^. According to the prevalence of gynecological cancers, the malignant tumor in the uterine corpus is the second leading cause of gynecological cancer in México^5^. The average ASMR from 2000 through 2022 was estimated to be 2.5, which is close to the worldwide estimated ASMR for 2019 (2.1; 95%CI 1.9–2.3), and a similar value is estimated for countries with a high sociodemographic index. Moreover, compared with other regions of Latin America, the Mexican ASMR was similar to that reported for Tropical Latin America (2.4; 95%CI 2.2–2.6) and Southern Latin America (2.4; 95%CI 2.2–2.6)^19^. Several risk factors have been associated with the development of malignant neoplasms of the corpus uteri.

However, obesity is one of the significant risk factors associated with malignant neoplasms of the corpus uteri. The increase in the incidence and prevalence of malignant neoplasms of the corpus uteri from 1990 to 2019 in regions with a high sociodemographic index is associated with an increase in the prevalence of obesity and insulin resistance and an increase in life expectancy^20^. Obesity is a pathological state characterized by increased estrogen levels caused by the aromatization of androgens (produced by suprarenal glands) by the adipose tissue. Furthermore, the hyperestrogenic state could induce the proliferation of endometrial tissue. Additionally, obesity is characterized by an increase in pro-inflammatory cytokines and adipokines that could be associated with an increase in proangiogenic and carcinogenic states^21^. A strong correlation has been reported between obesity and the incidence of endometrial cancer^22^. In this line, obesity shows an increase steadily from 2000 to 2018; the increase in the prevalence of obesity was calculated at 42.2%. Interestingly, Mexican women are more affected than men by obesity, and the risk for abdominal obesity increases with age^23^. A high correlation has been reported between obesity frequency and malignant neoplasms of the corpus uteri in a national reference hospital^24^. These facts could explain the increase in the ASMR associated with malignant neoplasms of the corpus uteri in México. More studies are necessary for describing the relationship between obesity and malignant neoplasms of the corpus uteri, focused on the impact of public health laws for decreasing obesity and its indirect effects on malignant neoplasms of corpus uteri prevalence.

In this study, the analysis of ASMR from malignant neoplasms of the corpus uteri by a federal entity showed that Chiapas and Guerrero are characterized by lower ASMR in comparison to the national average; in contrast, Ciudad de México showed higher ASMR compared to the national average. This result aligns with the 2022 crude death-related data for malignant tumors reported by the National Institute of Statistics, Geography, and Informatics (INEGI)^4^. In this line, Ciudad de México is the state with the highest frequency of obesity in México, and the sur mesoregions (that include Guerrero and Chiapas) reported a low frequency of obesity compared to the national average^25^. Moreover, life expectancy is different between federative entities. Ciudad de México reported the highest life expectancy compared to Guerrero and Chiapas^26^. Considering that malignant neoplasms of the corpus uteri are a disease intrinsically related to obesity and age, an increase in life expectancy and obesity frequency in Ciudad de México could be associated with the increase in ASMR from malignant neoplasms of the corpus uteri.

Age is a risk factor for the development of cancer in all mammals; two leading causes are proposed to explain this fact: the reduction in immune system capacity to detect and eliminate cancer cells and accumulative synergies between exposure to pro-oncogenic environmental factors and intrinsic individual factors^27^. The frequency distribution from cases of malignant neoplasms of the corpus uteri shows an increase in age≥35 years, with a peak at 60–64 years and a decrease in age higher than ≥65 years. Similar results are observed in malignant neoplasms of the corpus uteri mortality from the United States^28^ and the estimated global mortality attributed to malignant neoplasms of the corpus uteri^29^.

In clinical practice, postmenopausal women tend to reduce their gynecological checkups. Approximately 48% of postmenopausal women consider that postmenopausal bleeding is normal and, therefore, do not request medical attention. Postmenopausal bleeding is a common symptom of endometrial hyperplasia or cancer^30,31^. Nevertheless, the misconception that bleeding after menopause is normal could decrease the early detection of malignant neoplasms of the corpus uteri and increase the risk of mortality associated with late diagnosis. Our study results suggest the need to develop public health strategies to encourage the gynecological medical attention of postmenopausal women. The results also indicate that in the last 20 years, the mortality rate from malignant neoplasms of the corpus uteri in México has had a constant increase. Moreover, older women (60–65 years) are the most affected population. These findings highlight the need for new public health policies focused on reducing uterine cancer mortality.

Furthermore, the effect of the COVID-19 pandemic on changes in mortality rates from Mexico, including those related to malignant neoplasms of the corpus uteri, could be considered. Two COVID-19 high mortality rates were observed in Mexico in 2020; the first mortality spike occurred in June 2020 and the second in December of the same year. In the first spike, the states of Campeche, Tlaxcala, Quintana Roo, Tabasco, and Sonora were the most affected. In the second spike, CDMX, Baja California, Estado de México, and Chihuahua were the most affected states. During the COVID-19 pandemic, 26 states experienced an excess mortality rate of approximately 50% for 3 months, and seven states experienced an excess mortality rate of nearly 100%. Under this scenario, in 2020, the life expectancy decreased by 5.54 years^32^. Even though epidemic indicators have shown that Mexican men were more affected than women, Mexican women lost an average of 2.3 years of life expectancy due to COVID-19 in 2020. The impact of COVID-19 mortality in Mexican women was concentrated between the ages of 50 and 75 years, and the peak was registered in the 65- to 75-year-old age group. Notably, the age group of 60 to 65 years was most affected by malignant neoplasms of the corpus uteri^33^. The reduction in malignant neoplasms of corpus uteri mortality between 2020 and 2021 in Mexico could be associated with two key aspects observed during the COVID-19 pandemic: changes in public health services (several public hospitals were designated for attention specific to COVID-19 patients) and sub-register of causes of death (several deaths were erroneously attributed to COVID-19). In addition, a reduction of 5.3% of mortality associated with malignant neoplasms was observed in all death certificates during the COVID-19 pandemic^34^. The above information could be considered for interpreting the trends from malignant neoplasms of the corpus uteri in Mexico.

The legislation related to the prevention of malignant neoplasms of the corpus uteri in Mexico is in its infancy. In 2002, under the social program "IMSS-oportunidades," the gynecological care health services were promoted to the diagnosis and treatment of cervical cancer by early cytological diagnosis. In 2003, the public health care program "Seguro Popular" (Popular insurance) was established which provided free health services for the general population without social insurance. In this period, the "Seguro de gastos catastroficos" (Catastrophic expense insurance) program offered free medical care services for attending malignant neoplasms of the corpus uteri. By law, the accessibility to medical care services is free for women without social insurance diagnosed with malignant neoplasms of the corpus uteri. In 2011, the clinic guide for the Diagnosis and Treatment of Endometrial Cancer was implemented in the Mexican public health system. However, a specific law related to the prevention, diagnosis, and treatment of malignant neoplasms of the corpus uteri is nonexistent, in contrast with the law for breast cancer and cervical cancer, which shows the need for legislation of a new law for the prevention, diagnosis, and treatment of malignant neoplasms of the corpus uteri^35-38^.

The accuracy and completeness of death certificate register from Mexico are considered high. The quality assessment of the death certificate register in Mexico shows 100% completeness of registration, and the proportion of ill-defined codes is estimated at 3.8%^39^. Mexico's mortality statistics system establishes quality controls to ensure the reliability of the records. These include sequential reports by stage: during data capture, control figures are generated for data packages and sources; subsequently, a report detects potential duplicates. The capture inconsistencies phase allows for error correction, generating a list for deaths that validates the cause of death against the decedent's sex and age. Finally, simple frequencies are generated as an input for data review and release.

Furthermore, the non-response and non-coverage rate was estimated at 1.4 at the national level in 2023^40^. Other important issues to consider in light of the current results include the potential that the ICD-10 code C55 (Malignant Neoplasm of Uterus, Part Unspecified) may include records where precise anatomical and pathological identification and classification of the uterine neoplasm were not possible. As a result, this category could cover deaths that more accurately correspond to code C53 (Malignant Neoplasm of Cervix Uteri) or C54 (Malignant Neoplasm of Corpus Uteri). During the period from 2000 to 2022, the percentage distribution of deaths related to these three codes was as follows: C53 (82.26%), C54 (10.47%), and C55 (7.27%).
